# Association between the duration of smoking cessation and α−Klotho levels in the US middle-aged and elderly population

**DOI:** 10.1016/j.heliyon.2024.e38298

**Published:** 2024-09-24

**Authors:** Rui Du, Xiaoyan Tang, Cheng Yang, Jinhu Shi, Yuchen Lai, Shifang Ding, Wei Huang

**Affiliations:** a Department of Ultrasound, General Hospital of Central Theater Command, No.627, Wuluo Road, Wuhan 430070, Hubei, China; b Department of Cardiology, General Hospital of Central Theater Command, No.627, Wuluo Road, Wuhan 430070, Hubei, China; c Department of Radiology, General Hospital of Central Theater Command, No.627, Wuluo Road, Wuhan 430070, Hubei, China; d School of Medicine, Wuhan University of Science and Technology, No. 947, Heping Avenue, Wuhan 430065, Hubei, China

**Keywords:** Smoking cessation, α−Klotho, Aging, Life span, NHANES

## Abstract

**Background:**

α−Klotho is a molecule associated with aging and several diseases. Previous studies have reported decreased levels of serum α−Klotho (SαKl) in smokers compared to never smokers. Interestingly, we also found the SαKl level could partly recover in those who quit smoking. The objective of this study was to investigate SαKl levels in the US population who quit smoking for a certain period.

**Methods:**

A total of 9268 participants, ranging in age from 40 to 79 years were enrolled in this cross-sectional study, 37.04 % were identified as former smoker. Data from the NHANES conducted between 2007 and 2016 were utilized for analysis. The association between the period of smoking cessation and SαKl levels was evaluated through multivariate linear regression models. Additionally, a detailed analysis stratified by key clinical factors was performed.

**Results:**

The mean level of SαKl among the former smoker was 827.41 pg/mL. After full adjustment, the SαKl level increased over time after smoking cessation, with an increase of 1.20 pg/ml per year of abstinence (*P* = 0.005). The linear correlation persists regardless of the duration of the smoking habit before quitting. In the stratified analysis, a positive correlation was observed between duration of smoking cessation and SαKl levels in individuals aged 60–79 years, females, normal weight individuals, those involved in moderate or vigorous physical activity, and those with a history of cancer (all *P<*0.05).

**Conclusion:**

This study showed a positive association between the duration of smoking cessation and SαKl levels in former smokers. Prolonged abstinence may contribute to increased SαKl levels which may protect people against aging-related diseases.

## 1 Introduction

Smoking is a paramount public health challenge, responsible for over 200 million deaths in the last three decades [1]. Individuals who cease smoking exhibit significantly lower risk of cardiovascular disease (CVD), stroke, cancer, and mortality compared to persistent smokers [[2], [3], [4], [5]]. Meanwhile, smoking cessation can improve respiratory health, mental well-being, and life quality. It has been widely accepted that quitting smoking can reducing the mortality and morality of the chronic disease, improving the overall health status [6,7], and even extend lifespan for more than ten years [7]. Nevertheless, the specific mechanisms underlying the benefits of quitting smoking are still unclear.

α−Klotho (αKl), a transmembrane protein, is predominantly found in the distal tubules of the kidney [8]. It is essential for maintaining mineral balance, exerting anti-inflammatory and antioxidative effects, and mitigating senescence [8,9]. Smoking is a well-established risk factor for aging-related diseases, including CVD, cognitive impairment, and chronic kidney disease (CKD), all of which have also been linked to αKl dysregulation [[10], [11], [12]]. Cigarette smoking promotes oxidative stress [13] and chronic inflammation [14], two major pathways that contribute to both reduced αKl expression and the pathogenesis of these diseases [9]. Our previous study focused on the immediate association between smoking status and serum α−Klotho (SαKl) levels. We found that current smoking was associated with decreased SαKl levels, suggesting a negative impact of ongoing smoking on SαKl. In contrast, we observed no significant difference in SαKl levels between former and never-smokers [15]. It remains unclear whether an extended period of abstinence from smoking, typically spanning several years to even decades, might gradually lead to increased αKl levels, potentially reversing some of the damage caused by smoking.

In this study, we hypothesize that the duration of smoking cessation is positively associated with SαKl levels in former smokers. By conducting subgroup analyses of age, gender and other factors. We designed the study to explore the relationship between the duration of smoking cessation and SαKl.

## 2 Materials and methods

### 2.1 Study population

The study utilized data from the NHANES, a comprehensive initiative spearheaded by the US Centers for Disease Control and Prevention. NHANES employs a stratified, multistage sampling technique to represent the demographic composition of the US civilian population. For this study, we included 50,588 subjects from five consecutive NHANES survey cycles conducted between 2007 and 2016. This period and age range (40−79 years) were specifically chosen because SαKl levels were measured only during this time and within this age group. We suppose the level of SαKl may be more widely concerned among this group of people. Meanwhile, the level of SαKl is more closely related to diseases and less affected by being too young or too old in this group of people (middle-aged adult) [16,17]. From the initial sample, 31,244 individuals were excluded due to age criteria. Additionally, 5580 participants with missing data on SαKl, 9 pregnant participants, 64 participants with missing smoking data, and 2697 current smokers were excluded. Furthermore, 1726 participants were excluded due to missing data on other relevant variables. After applying these criteria, the final analysis included data from 9268 participants (Fig. S1).

### 2.2 Smoking status

Standardized questionnaires were employed during interviews to ascertain the smoking status of participants. These questionnaires were designed to gather detailed information about participants' smoking history. Specifically, individuals were inquired about whether they had smoked a cumulative total of at least 100 cigarettes throughout their lifetime and if they were current smokers. Based on their responses, participants were stratified into three distinct categories: nonsmokers, former smokers, and current smokers. Individuals who had smoked less than 100 cigarettes in their lifetime were categorized as nonsmokers, also referred to as 'never smokers'. Those who had smoked over 100 cigarettes but had quit by the survey date were defined as former smokers. Current smokers were identified as those who had smoked more than 100 cigarettes and continued to smoke at the time of the survey.

Participants were identified as former smokers if they had not smoked any cigarettes since their reported quit date, and the elapsed time since quitting smoking was meticulously calculated and transformed into a variable labeled 'years since cessation.' This variable served as a critical exposure factor within the analytical framework of our study. To facilitate a nuanced analysis, the period since discontinuing smoking was segmented into four categories: under 5 years, between 5 and 10 years, between 10 and 20 years, and more than 20 years. This categorization allowed for a granular examination of the potential health impacts associated with varying durations of smoking cessation.

### 2.3 Serum levels of α−Klotho

The assessment of SαKl levels in the frozen serum specimens collected during the NHANES 2007–2016 cycle was conducted following a comprehensive evaluation of laboratory methodologies and protocols in 2019–2020, as outlined in the NHANES Laboratory/Medical Technologists Procedures Manual [18]. Specimens were meticulously preserved at an ultra-low temperature of −80 °C to ensure their integrity until analysis. The samples were fresh-frozen and analyzed using pristine serum from participants aged 40–79. All analyses were conducted at the Northwest Lipid Metabolism and Diabetes Research Laboratories, University of Washington, where strict laboratory protocols were followed to maintain sample quality. The quantification of SαKl levels was performed utilizing an enzyme-linked immunosorbent assay kit, developed explicitly for this purpose by IBL International, Japan. Adherence to the manufacturer's guidelines was stringently observed throughout the analytical process.

To enhance the reliability of the results, each serum sample underwent duplicate analyses. The final reported value for each specimen was established by calculating the mean of the two individual measurements, thereby mitigating potential discrepancies between them. The sensitivity threshold of the assay was identified at 6.0 pg/mL, ensuring the capability to detect even minimal concentrations of SαKl. Across the studied cohort, the mean SαKl concentration was reported at 698.0 pg/mL, with a broad range spanning from 285.8 to 1638.6 pg/mL, reflecting the variability in α-Klotho levels among participants [19].

### 2.4 Other covariates

In our analysis, we meticulously evaluated a range of potential confounding variables, encompassing age, race/ethnicity (Mexican American, other Hispanic, non-Hispanic White, non-Hispanic Black, and Others), body mass index (BMI) classified as Normal weight (<25 kg/m^2^), overweight (25–30 kg/m^2^), and obese (≥30 kg/m^2^), the poverty income ratio (PIR) segmented into three groups (<1.30, 1.30−2.99, and ≥3.00), levels of education (less than high school, high school or GED, above high school), and physical activity (PA) levels (inactive, moderate, or vigorous). Additionally, alcohol consumption was categorized based on previously published criteria [20] (never drinker, former drinker, light-to-moderate drinker, heavy drinker).

Regarding comorbidities within the scope of our study, we considered diabetes, hypertension, CVD, and cancer. Participants were identified as diabetic based on several criteria: a diagnosis of diabetes, current diabetes usage, or specific glucose levels—HbA1c levels ≥6.5 %, fasting serum glucose ≥7.0 mmol/L, or random plasma glucose ≥11.1 mmol/L. Hypertension status was assigned to participants showing a mean systolic blood pressure of 140 mmHg or higher, diastolic blood pressure of 90 mmHg or higher, or if they were regularly taking antihypertensive medication. The identification of CVD or cancer relied upon a physician's diagnostic confirmation.

### 2.5 Statistical analysis

Procedures were implemented in this study to incorporate multiple years of NHANES data (10 years). The analysis was weighted according to NHANES weighting guidelines to produce estimates that are representative at a national level. To assess baseline characteristics, weighted linear regression was used for continuous variables and weighted chi-square tests for categorical variables. The findings were presented as weighted percentages (95 % Conﬁdence interval) for categorical data and weighted means ± Standard Error (SE) for continuous data. Multivariate linear regression models were utilized to estimate the relationship between the duration of smoking cessation and SαKl levels. The β coefficients were calculated to quantify the effect size of smoking cessation duration on SαKl levels, estimating the change in SαKl levels per unit increase in smoking cessation duration.

Three multivariate models were developed following the Strengthening the Reporting of Observational Studies in Epidemiology (STROBE) guidelines. The models were adjusted for age, gender, and race/ethnicity (Model 1). Additionally, adjustments were made for BMI, marital status, education level, PIR, alcohol consumption status, and PA (Model 2). Furthermore, adjustments were made for eGFR, diabetes, hypertension, CVD, and cancer (Model 3). The smoking cessation time was treated as a continuous independent and categorical variable, with less than 5 years as the reference category. To explore the potential nonlinear association between the duration of smoking cessation and SαKl, generalized additive models and restricted cubic splines (smooth curve fittings, RCS) were used.

Additionally, stratiﬁed analyses were conducted to examine the relationship between the duration of smoking cessation and SαKl in different subgroups, including age (</≥ 60 years), gender, BMI, PA, and comorbidities. Sensitivity analyses included the total number of years of smoking among former smokers in Model 3. The correlations between the duration of smoking cessation and SαKl levels were analyzed using an RCS. A 2-tailed *P* < 0.05 was used to determine statistical significance. The analyses were performed using R software (version 4.3.2) and Free Statistics software (version 1.9).

## 3 Results

### 3.1 Population characteristics

Our research enrolled in a total of 9268 adults aged over 40 years old (including 40 years old), representing a weighted population of 79,083,669. Among these individuals, 3419 adults (weighted 37.04 %) were identified as former smokers. The mean age of the former smokers was 59.1 ± 0.3 years, 55.63 % were males, and the mean level of SαKl was 827.41 ± 7.26 pg/mL. The demographic and health characteristics of the sample were segregated according to smoking status (in Table 1).

**Table 1:** Survey-weighted characteristics of the overall NHANES sample according to duration of smoking cessation.

Characteristic	Total	Never smoking	Duration of smoking cessation				*P*
			<5 years	5–10 years	10–20 years	≥20 years	
Age, years	56.8 ± 0.2	55.4 ± 0.2	54.2 ± 0.4	56.5 ± 0.6	56.7 ± 0.5	63.2 ± 0.4	<0.0001
α-klotho, pg/mL	849.46 ± 5.77	862.44 ± 6.73	833.29 ± 15.53	803.66 ± 15.80	829.06 ± 13.87	830.76 ± 10.77	<0.001
eGFR, mL/min/1.73m^2^	85.53 ± 0.36	86.52 ± 0.42	88.22 ± 1.11	86.10 ± 1.07	87.03 ± 0.78	79.61 ± 0.57	<0.0001
Quit time, years	19.56 ± 0.36		2.30 ± 0.13	8.46 ± 0.09	16.14 ± 0.15	32.00 ± 0.25	<0.0001
**Gender (%)**							<0.0001
Female	52.61 (48.38,56.84)	57.46 (55.99,58.93)	45.76 (40.13,51.39)	45.74 (39.14,52.34)	44.66 (39.85,49.47)	43.22 (39.91,46.54)	
Male	47.39 (43.61,51.17)	42.54 (41.07,44.01)	54.24 (48.61,59.87)	54.26 (47.66,60.86)	55.34 (50.53,60.15)	56.78 (53.46,60.09)	
**Race/ethnicity (%)**							<0.0001
Mexican American	6.37 (5.03,7.70)	6.93 (5.21,8.65)	7.11 (5.01,9.21)	7.09 (4.92,9.27)	5.91 (4.03,7.79)	3.94 (2.81,5.07)	
Other Hispanic	4.36 (3.44,5.28)	4.71 (3.61,5.80)	4.62 (2.76,6.48)	4.17 (2.53,5.81)	4.02 (2.71,5.33)	3.15 (2.20,4.09)	
Non-Hispanic White	75.49 (67.18,83.80)	72.73 (69.51,75.95)	76.41 (71.39,81.43)	76.84 (71.87,81.81)	77.35 (73.41,81.28)	84.30 (81.83,86.77)	
Non-Hispanic Black	8.05 (7.02,9.09)	9.05 (7.57,10.52)	6.84 (4.99,8.69)	7.59 (5.33,9.85)	7.78 (5.95,9.61)	5.06 (3.79,6.33)	
Others	5.73 (4.89,6.57)	6.59 (5.50,7.69)	5.02 (2.95,7.10)	4.31 (2.20,6.41)	4.94 (2.94,6.94)	3.56 (2.46,4.65)	
**BMI (%)**							0.01
Normal weight	22.07 (19.78,24.35)	23.79 (22.19,25.38)	17.27 (13.38,21.15)	16.02 (12.30,19.73)	19.29 (15.27,23.30)	20.76 (17.48,24.05)	
Overweight	35.66 (32.43,38.89)	35.79 (34.10,37.49)	35.85 (30.15,41.55)	36.93 (31.65,42.20)	34.49 (29.85,39.13)	35.34 (32.08,38.60)	
Obese	42.28 (38.83,45.72)	40.42 (38.35,42.50)	46.89 (41.56,52.21)	47.06 (41.27,52.85)	46.22 (40.91,51.54)	43.90 (40.14,47.65)	
**Marital status (%)**							<0.001
Married/living with partner	72.93 (66.51,79.35)	73.58 (71.84,75.32)	63.58 (59.02,68.15)	69.99 (64.15,75.83)	71.21 (67.16,75.26)	76.25 (72.73,79.76)	
Living alone	27.07 (25.25,28.89)	26.42 (24.68,28.16)	36.42 (31.85,40.98)	30.01 (24.17,35.85)	28.79 (24.74,32.84)	23.75 (20.24,27.27)	
**Education level (%)**							<0.0001
Less than high school	13.33 (11.88,14.78)	12.02 (10.41,13.64)	17.69 (14.01,21.37)	22.97 (17.97,27.97)	17.01 (13.27,20.75)	11.73 (9.88,13.58)	
High school or GED	20.41 (18.19,22.64)	19.21 (17.61,20.81)	22.47 (17.48,27.46)	23.15 (18.06,28.25)	26.11 (21.63,30.60)	20.26 (17.18,23.33)	
Above high school	66.26 (60.32,72.19)	68.76 (66.23,71.30)	59.84 (54.01,65.67)	53.88 (47.60,60.15)	56.88 (51.23,62.53)	68.01 (64.42,71.60)	
**PIR (%)**							<0.0001
<1.30	13.81 (12.43,15.20)	13.84 (12.21,15.47)	19.60 (15.50,23.71)	13.38 (10.76,16.01)	16.74 (13.56,19.91)	9.75 (7.58,11.92)	
1.30−2.99	25.08 (22.62,27.54)	23.73 (21.80,25.66)	27.36 (23.12,31.60)	32.23 (26.32,38.13)	28.76 (23.72,33.80)	25.23 (21.48,28.98)	
≥3.00	61.11 (54.91,67.31)	62.43 (59.46,65.40)	53.04 (47.68,58.40)	54.39 (48.05,60.73)	54.51 (48.88,60.14)	65.02 (60.46,69.57)	
**Alcohol consumption (%)**							<0.0001
Never drinker	11.86 (10.47,13.26)	17.06 (15.06,19.07)	2.52 (1.38,3.66)	3.36 (1.50,5.22)	2.99 (2.04,3.94)	3.15 (2.07,4.22)	
Former drinker	17.16 (15.54,18.78)	13.75 (12.56,14.94)	20.81 (16.31,25.30)	26.28 (19.47,33.10)	23.45 (19.35,27.55)	22.67 (19.10,26.23)	
Light-to-moderate drinker	59.75 (54.00,65.49)	59.36 (56.61,62.10)	52.21 (47.45,56.96)	51.44 (43.83,59.05)	62.26 (57.46,67.05)	65.50 (61.46,69.54)	
Heavy drinker	11.23 (10.09,12.37)	9.83 (8.87,10.79)	24.47 (20.28,28.65)	18.91 (14.50,23.32)	11.30 (8.19,14.42)	8.69 (6.64,10.73)	
**Physical activity (%)**							<0.0001
Inactive	45.53 (41.52,49.54)	44.44 (41.68,47.20)	50.51 (44.82,56.20)	56.84 (50.71,62.97)	49.67 (43.70,55.63)	42.11 (38.41,45.82)	
Moderate	33.29 (29.89,36.68)	32.12 (30.16,34.08)	32.96 (27.94,37.98)	25.46 (19.94,30.97)	30.50 (25.28,35.71)	41.61 (37.51,45.72)	
Vigorous	21.18 (18.53,23.84)	23.44 (21.18,25.70)	16.53 (11.84,21.22)	17.70 (11.94,23.46)	19.83 (15.03,24.64)	16.27 (13.28,19.27)	
**Diabetes (%)**							<0.0001
No	80.05 (73.18,86.92)	82.16 (80.68,83.63)	75.88 (71.62,80.14)	77.52 (72.84,82.21)	76.85 (73.58,80.12)	76.22 (73.09,79.35)	
Yes	19.95 (18.34,21.56)	17.84 (16.37,19.32)	24.12 (19.86,28.38)	22.48 (17.79,27.16)	23.15 (19.88,26.42)	23.78 (20.65,26.91)	
**Hypertension (%)**							<0.0001
No	51.45 (46.90,56.00)	54.77 (52.88,56.65)	50.98 (45.23,56.73)	45.83 (38.81,52.86)	47.35 (42.20,52.50)	42.75 (39.18,46.32)	
Yes	48.55 (44.81,52.30)	45.23 (43.35,47.12)	49.02 (43.27,54.77)	54.17 (47.14,61.19)	52.65 (47.50,57.80)	57.25 (53.68,60.82)	
**CVD (%)**							<0.0001
No	89.82 (82.65,96.99)	92.60 (91.77,93.42)	87.15 (84.01,90.28)	82.98 (77.74,88.22)	85.31 (82.56,88.06)	84.70 (82.64,86.75)	
Yes	10.18 (9.24,11.13)	7.40 (6.58, 8.23)	12.85 (9.72,15.99)	17.02 (11.78,22.26)	14.69 (11.94,17.44)	15.30 (13.25,17.36)	
**Cancer (%)**							<0.0001
No	85.78 (79.14,92.42)	88.09 (87.13,89.05)	89.81 (86.72,92.91)	84.38 (80.20,88.55)	82.28 (78.19,86.36)	77.49 (74.26,80.71)	
Yes	14.22 (12.84,15.59)	11.91 (10.95,12.87)	10.19 (7.09,13.28)	15.62 (11.45,19.80)	17.72 (13.64,21.81)	22.51 (19.29,25.74)	

Note. Mean ± SE for continuous variables: *P* value for weighted linear regression model. % (95 % Conﬁdence interval CI) for categorical variables: *P* value for weighted chi-square test.

**Abbreviations:** BMI, Body Mass Index; PIR, Poverty income ratio; CKD, Chronic kidney disease; CVD, Cardiovascular disease.

It was observed that individuals who had successfully quit smoking for a longer duration tended to be older, more likely to be male, Non-Hispanic White, married or living with a partner, and normal weight (in Table 1). Furthermore, this group showed higher PIR, engaged in moderate physical activity, had a lower ratio of them with heavy alcohol consumption and experienced a greater prevalence of comorbid conditions.

### 3.2 Univariate analysis of SαKl levels

A univariate analysis was performed to identify the factors that might impact the levels of SαKl (Table S1). The analysis revealed that age, gender, race/ethnicity, BMI, marital status, alcohol consumption, eGFR, hypertension, CVD, and cancer were significantly associated with SαKl levels. However, there was no statistical significance in SαKl levels (*P* = 0.64) among the population with different durations of smoking cessation.

### 3.3 The SαKl levels of never smokers and former smokers

After adjusting for demographic factors in Model 1, SαKl levels were significantly lower in former smokers compared never smokers (β = −18.40; 95 % CI: −33.32, −3.49; *P* = 0.018) as shown in Table 2. However, in Model 2 and Model 3, which accounted for additional confounding variables, the differences in SαKl levels between former smokers and never smokers were not statistically significant (β = −9.89; 95 % CI: −26.19, 6.41; *P* = 0.239; β = −12.08; 95 % CI: −27.79, 3.62; *P* = 0.137, respectively) as shown in Table 2.

**Table 2:** The serum α−klotho levels of never smokers and former smokers.

	*n*	Model 1	*P*	Model 2	*P*	Model 2	*P*
		β (95 % CI)		β (95 % CI)		β (95 % CI)	
Never smokers	5849	Ref		Ref		Ref	
Former smokers	3419	−18.40 (−33.32∼−3.49)	0.018	−9.89 (−26.19–6.41)	0.239	−12.08 (−27.79–3.62)	0.137

Model 1: Adjusted for age, gender, and race/ethnicity.

Model 2: Adjusted for the items in Model 1 + BMI, marital status, PIR, education level, alcohol consumption, and PA.

Model 3: Adjusted for the items Model 2 + diabetes, hypertension, eGFR, CVD, and cancer.

**Abbreviations:** BMI, Body mass index; PIR, Poverty income ratio; PA, Physical activity; CVD, Cardiovascular disease.

### 3.4 Relationship between the duration of smoking cessation and serum α−Klotho levels

We investigated in the correlation between the duration of smoking cessation and the SαKl levels in individuals who had previously smoked (Table 3). By adjusting pivotal demographic factors in Model 1, the levels of SαKl increased over time after smoking cessation (*β* = 1.21; 95 % CI: 0.39, 2.03; *P* = 0.004). The significance was consistent in Model 2 and Model 3 (*β* = 1.14; 95 % CI: 0.30, 1.98; *P* = 0.008; *β* = 1.20; 95 % CI: 0.37, 2.04; *P* = 0.005, respectively). Which means that SαKl levels increased by 1.20 pg/ml for each additional year abstained from smoking in the smoking cessation group.

**Table 3:** Associations between the duration of smoking cessation and serum α−klotho levels.

	Years since quitting									*Trend test*	*P* for Trend
	Continuous	*P*	<5 years	5–10 years,	*P*	10–20 years,	*P*	>20 years,	*P*		
				β (95 % CI)		β (95 % CI)		β (95 % CI)			
*n*	*3419*		650	454		841		1474		*3419*	
**Model 1**	1.21 (0.39–2.03)	0.004	Ref.	−20.25 (−55.38–14.89)	0.259	6.04 (−23.69–35.77)	0.690	34.47 (6.52–62.43)	0.016	13.80 (4.89–22.70)	0.002
**Model 2**	1.14 (0.30–1.98)	0.008	Ref.	−23.21 (−58.41–11.98)	0.196	0.71 (−29.20–30.62)	0.963	30.77 (2.35–59.19)	0.034	12.51 (3.43–21.60)	0.007
**Model 3**	1.20 (0.37–2.04)	0.005	Ref.	−20.87 (−55.80–14.05)	0.242	1.27 (−28.43–30.97)	0.933	34.48 (6.24–62.72)	0.017	13.56 (4.53–22.59)	0.003

Note: 95%CI, 95 % Confidence interval.

Model 1: Adjusted for age, gender, and race/ethnicity.

Model 2: Adjusted for the items in Model 1 + BMI, marital status, PIR, education level, alcohol consumption, and PA.

Model 3: Adjusted for the items Model 2 + diabetes, hypertension, eGFR, CVD, and cancer.

**Abbreviations:** BMI, Body mass index; PIR, Poverty income ratio; PA, Physical activity; CVD, Cardiovascular disease.

**Fig. 1:**
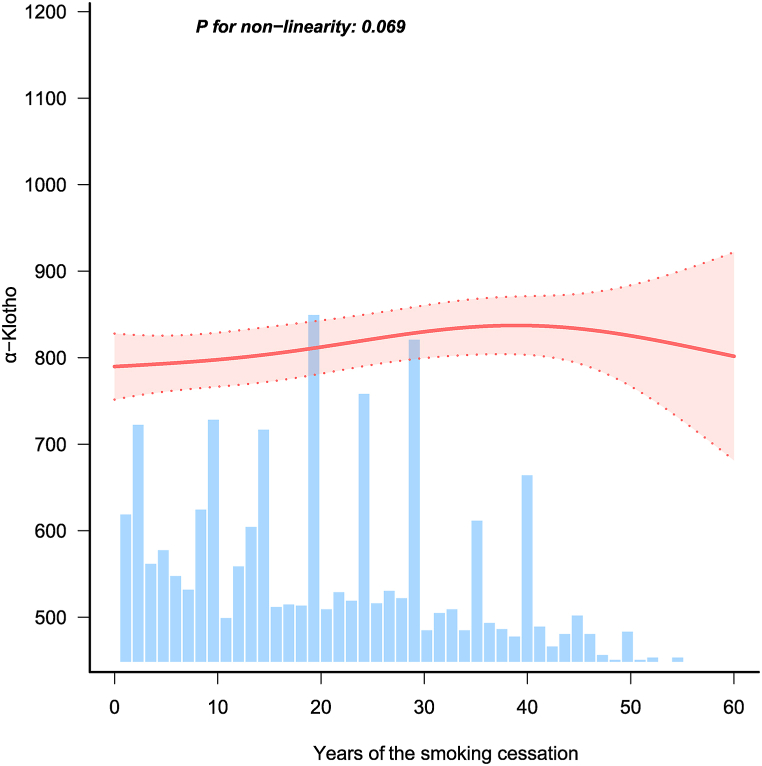
Correlation between the duration of smoking cessation and α−Klotho levels by smooth curve fittings.

When classified by the duration of smoking cessation (shown in Models 1, 2, and 3), individuals who quit smoking for over 20 years exhibited higher SαKl levels than those who had quit for less than 5 years (All *P* for trend <0.01). Meanwhile, a linear positive correlation between the duration of smoking cessation and SαKl was found by RCS analysis (*P*-nonlinear = 0.069, Fig. 1).

### 3.5 Subgroup, sensitivity, and additional analysis

**Fig. 2:**
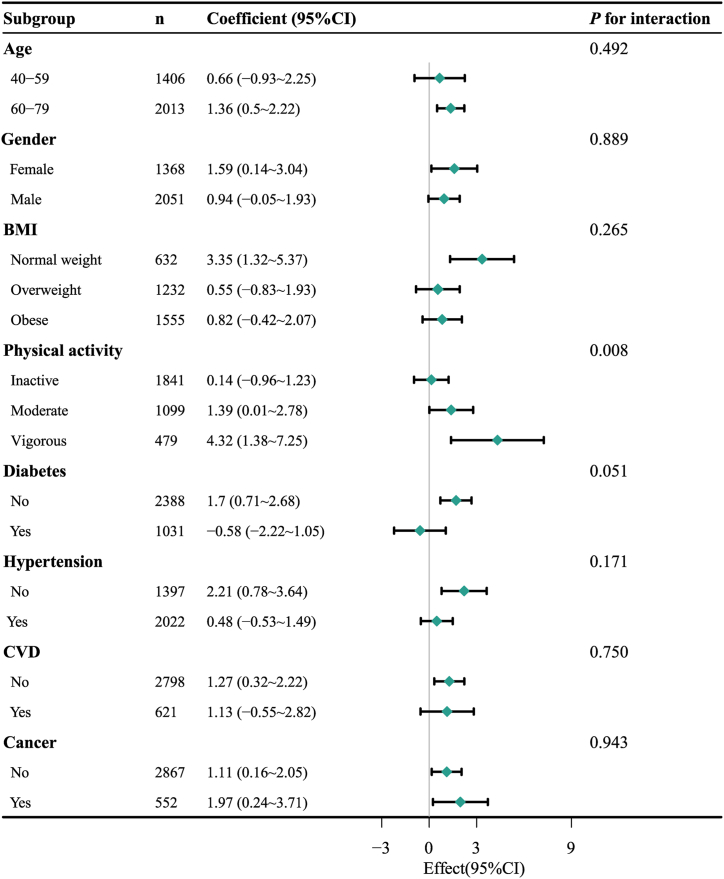
Subgroup analyses of the association between the duration of smoking cessation and serum α−Klotho levels. Adjusted for age, gender, race/ethnicity, BMI, marital status, PIR, education level, alcohol consumption, physical activity, diabetes, hypertension, eGFR, CVD, and cancer. Abbreviations: BMI, body mass index; PIR, ratio of family income to poverty; CVD, cardiovascular disease.

In stratified analyses, the positive correlation between the duration of smoking cessation and SαKl levels remained consistent in the subgroups of people aged 60–79 years, with female gender, normal weight, did moderate or vigorous physical activity, and had personal cancer history (Fig. 2). However, this positive correlation was not found to be significant among individuals with diabetes, hypertension, or CVD. A significant interaction was observed between the duration of smoking cessation and SαKl levels in participants with different degrees of physical activity (*P* = 0.008). It was found that SαKl levels were notably higher in active participants who engaged in moderate and vigorous physical activity, whereas they remained unchanged in inactive participants.

Furthermore, in another sensitivity analysis, the total smoking duration of the former smokers before quitting was added in model 3, and the results remained consistent with the unadjusted data (Table S2).

## 4 Discussion

Some studies have shown that the mortality risk of former smokers gradually decreases over time [21]. Quitting smoking can bring significant health benefits and lower down the All-Cause Mortality [[22], [23], [24]]. This study investigated the correlation between the duration of smoking cessation and levels of SαKl (a protein involved in aging and various biological processes). Our findings indicate the levels of SαKl increased in a time-dependent manner (the duration of the time quitted smoking) among individuals who quit smoking.

Previous studies on the association between smoking and SαKl levels remained controversial. Some early studies with small sample sizes reported higher SαKl levels in habitual smokers [25,26]. Kamizono et al. observed a decrease in SαKl levels shortly after smoking cessation in a study involving 28 participants [27]. However, our previous research has found that habitual smokers had significantly lower SαKl levels compared to people who never smoked [15]. In this study, we observed that the increase in SαKl was linearly correlated with the duration of smoking cessation, and this correlation persisted regardless of the duration of the smoking habit before quitting.

The rise in SαKl levels in former smokers who quit for a certain duration may be attributed to several factors. Firstly, smoking induces significant oxidative stress [28], generating excessive free radicals and reactive oxygen species (ROS), which damage kidney cells [29] and directly reduce SαKl synthesis and secretion. After cessation, the body's antioxidative capacity gradually recovers [30,31], potentially leading to a rise in SαKl as assists in repairing and mitigating oxidative damage. Secondly, the relief of chronic inflammation following smoking cessation may contribute to increased SαKl levels [32]. SαKl, an anti-inflammatory protein, rises as the body recovers [33], which was enhanced by extending the duration of smoking cessation. Thirdly, smoking causes endothelial dysfunction [34]. Quitting smoking upregulates SαKl levels which may gradually improve endothelial function [35] and strengthen protective mechanisms, reflecting overall cardiovascular recovery and long-term health benefits.

Besides, we observed significantly higher SαKl levels in participants who engaged in moderate and vigorous physical activity. Physical activity enhances antioxidative and anti-inflammatory responses [36] which may account for the upregulation of SαKl. Additionally, participants with normal BMI had higher SαKl levels compared to overweight and obese individuals. Obesity triggers systemic inflammation and oxidative stress [37,38], potentially diminishing the benefits of smoking cessation on SαKl levels. Lack of exercise can lead to weight gain and obesity [39], which may offset the SαKl increase induced by smoking cessation. Further research is needed to clarify the complex interplay between obesity, smoking cessation and SαKl levels. These findings underscore the importance of combining physical activity with cessation strategies to optimize the health benefits of smoking control policies.

Surprisingly, the benefit of smoking cessation assessed by SαKl levels shows no significance in individuals with diabetes, hypertension, or CVD. This suggests that SαKl levels may be more influenced by the severity of these chronic diseases than by smoking habit [[40], [41], [42]]. Therefore, the impact of smoking cessation on SαKl is minor compared to these conditions. However, there was a positive association between smoking cessation duration and SαKl levels in cancer patients, which may be related to a higher willingness to quit smoking. Nevertheless, studies showed that long term smoking cessation reduces the risk of hypertension, type 2 diabetes, CKD, and prevent their complications [[43], [44], [45]]. Therefore, these findings do not conclusively indicate that quitting smoking in patients with chronic diseases is without benefits.

The more significant correlation between the duration of smoking cessation and the increase in SαKl levels in females but not in males may be explained by several factors. In humans, females have longer telomeres and greater stem cell regeneration and proliferation [46]. In animal models, healthy female mice have fewer senescent cells (at both 4–5 months of age and 30 months of age) [47] and superior proteosomes activity, which is crucial for maintaining cellular homeostasis by removing defective proteins [48], compared to males. Additionally, estrogen has been shown to modulate αKl in rat hippocampal neurons, both in vivo and in vitro, enhancing the presynaptic vesicular glutamate transporter 1-positive clusters along dendrites [49]. These factors may contribute to the observed differences in SαKl levels between females and males post-smoking cessation.

Our results reveal that SαKl levels are elevated in former smokers when quitting, implying that quitting smoking may bring benefits by affecting SαKl. The underlying mechanism may refer to the recovery of the SαKl by alleviating the inflammatory conditions, oxidative stress and et al., which were supposed to anti-age and maintain a better overall state of health. Nevertheless, further research is needed to substantiate this hypothesis.

One of the primary strengths of this study is its reliance on a nationally representative sample, enhancing the generalizability of the findings. The study also controlled for potential confounding variables across socio-demographic and health behavior factors using the comprehensive NHANES dataset. However, there are several limitations. First, self−reporting data may introduce bias, leading to discrepancies with the actual status. Second, as a cross-sectional study, it cannot establish temporality, leaving the direction of the relationship between the duration of smoking cessation and SαKl levels unclear and raising the risk of reverse causation. Third, information regarding the duration and sources of secondhand smoke exposure is not provided in the NHANES dataset. Fourth, despite substantial adjustments for confounders, the possibility of residual, unmeasured, or unknown confounding remains, a common challenge in observational research. Lastly, some mechanisms proposed in our study are supported only by literature, and further laboratory studies are needed to confirm their reliability.

## 5 Conclusions

Our study revealed a considerable correlation between the duration of smoking cessation and serum α−Klotho levels, especially noticeable in individuals with a normal BMI, active physical activity, and females. These findings underscore the potential anti-aging benefits of smoking cessation, given Klotho's possible role in enhancing resistance to oxidative stress. Despite residual unmeasured or unknown confounding factors, our findings contribute valuable insights into the public health realm, underlining the multifaceted health benefits of quitting smoking.

## Data availability

Data used for this study are available on the NHANES website: https://wwwn.cdc.gov/nchs/nhanes/.

## Ethics approval and consent to participate

Documented, signed consent was obtained from all participants in the NHANES studies spanning 2007 to 2016. Approval for these studies was granted by the ethics review board at the 10.13039/100005262NCHS.

## CRediT authorship contribution statement

**Rui Du:** Writing – original draft, Software, Methodology, Investigation, Formal analysis, Data curation, Conceptualization. **Xiaoyan Tang:** Writing – original draft, Software, Methodology, Investigation, Data curation, Conceptualization. **Cheng Yang:** Validation, Methodology, Formal analysis, Data curation. **Jinhu Shi:** Visualization, Software, Methodology, Formal analysis. **Yuchen Lai:** Visualization, Validation, Data curation. **Shifang Ding:** Writing – review & editing, Supervision, Project administration, Conceptualization. **Wei Huang:** Writing – review & editing, Writing – original draft, Visualization, Project administration, Conceptualization.

## Declaration of competing interest

The authors declare that they have no known competing financial interests or personal relationships that could have appeared to influence the work reported in this paper.

## Abbreviations

αKl: α−Klotho
BMI: Body Mass Index
CI: Conﬁdence interval
CKD: Chronic kidney disease
CVD: Cardiovascular disease
NCHS: National Center for Health Statistics
NHANES: National Health and Nutrition Examination Survey
PIR: Poverty income ratio
ROS: Reactive oxygen species
PA: Physical activity
RCS: Smooth curve fittings
SαKl: Serum α−Klotho
