# Supplementary figures and images for: Association between the duration of smoking cessation and α−Klotho levels in the US middle-aged and elderly population
Source: Heliyon. 2024 Sep 24;10(19):e38298. doi: 10.1016/j.heliyon.2024.e38298 (PMC11467537; doi:10.1016/j.heliyon.2024.e38298)

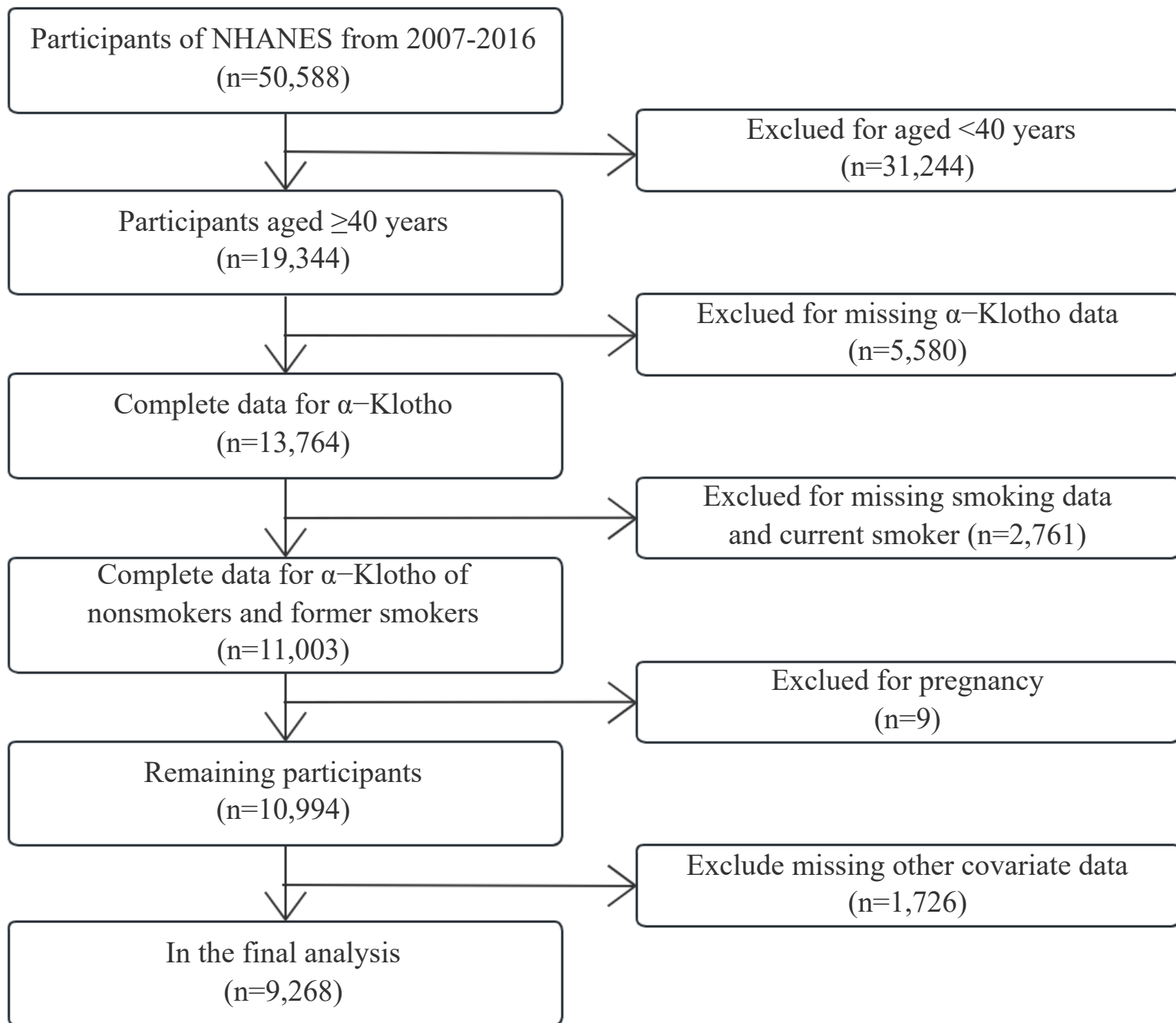

Supplement: Multimedia component 1 [file mmc1.pdf]
