# Supplementary material for: Association between the duration of smoking cessation and α−Klotho levels in the US middle-aged and elderly population
Source: Heliyon. 2024 Sep 24;10(19):e38298. doi: 10.1016/j.heliyon.2024.e38298 (PMC11467537; doi:10.1016/j.heliyon.2024.e38298)
Supplement: Multimedia component 2 [file mmc2.docx]

**Table S1.** Univariate analysis for serum α-klotho level (pg/ml)

|  | **β (95% CI)** | ***P*** |
| --- | --- | --- |
| Age | -2.68(-3.41, -1.96) | <0.0001 |
| eGFR | 2.22(1.83, 2.60) | <0.0001 |
| Quit years | -0.22(-1.15, 0.71) | 0.64 |
| **Gender** |  |  |
| Female | Ref | Ref |
| Male | -37.94(-55.66, -20.22) | <0.0001 |
| **Race/ethnicity** |  |  |
| Mexican American | Ref | Ref |
| Other Hispanic | 26.5(-5.96, 58.95) | 0.11 |
| Non-Hispanic White | -15.94(-35.39, 3.52) | 0.11 |
| Non-Hispanic Black | 75(42.19, 107.82) | <0.0001 |
| Others | 14.93(-17.59, 47.44) | 0.36 |
| **BMI** |  |  |
| Normal weight | Ref | Ref |
| Overweight | -32.12(-57.16, -7.08) | 0.01 |
| Obese | -36.96(-58.37, -15.55) | <0.001 |
| **Marital status** |  |  |
| Married/living with partner | Ref | Ref |
| Living alone | 20.75(4.85, 36.65) | 0.01 |
| **Education level** |  |  |
| Less than high school | Ref | Ref |
| High school or GED | -23.84(-51.39, 3.70) | 0.09 |
| Above high school | 3.99(-19.66, 27.64) | 0.74 |
| **PIR** |  |  |
| <1.30 | Ref | Ref |
| 1.30-2.99 | -7.79(-28.06, 12.49) | 0.45 |
| ≥3.00 | -8.26(-26.74, 10.22) | 0.38 |
| **A****lcohol consumption** |  |  |
| Never drinker | Ref | Ref |
| Former drinker | -38.66( -66.47, -10.84) | 0.01 |
| Light-to-moderate drinker | -42.52( -66.05, -19.00) | <0.001 |
| Heavy drinker | -77.32(-111.32, -43.32) | <0.0001 |
| **Physical activity** |  |  |
| Inactive | Ref | Ref |
| Moderate | -15.78(-33.63, 2.07) | 0.08 |
| Vigorous | 20.97( -3.81, 45.74) | 0.10 |
| Diabetes | -7.55(-26.20, 11.10) | 0.42 |
| Hypertension | -24.24(-38.78, -9.70) | 0.001 |
| CVD | -33.78(-56.44, -11.12) | 0.004 |
| Cancer | -41.27(-60.24, -22.30) | <0.0001 |

**Abbreviations:** BMI, Body Mass Index; PIR, Ratio of family income to poverty; CKD, chronic kidney disease; CVD, cardiovascular disease.
