# Supplementary material for: Association between the duration of smoking cessation and α−Klotho levels in the US middle-aged and elderly population
Source: Heliyon. 2024 Sep 24;10(19):e38298. doi: 10.1016/j.heliyon.2024.e38298 (PMC11467537; doi:10.1016/j.heliyon.2024.e38298)
Supplement: Multimedia component 3 [file mmc3.docx]

**Table S2.** Associations between smoking cessation and serum α−klotho levels.

|  | **Years since quitting**  ***))*** | | | | | | | | |  |  |
| --- | --- | --- | --- | --- | --- | --- | --- | --- | --- | --- | --- |
|  | **Continuous** | ***P*** | **<5 years** | **5-10 years, β (95% CI)** | ***P*** | **10-20 years, β (95% CI)** | ***P*** | **>20 years, β (95% CI)** | ***P*** | ***Trend test*** | ***P* for Trend** |
| **n** | ***3419*** |  | **650** | **454** |  | **841** |  | **1474** |  | ***3419*** |  |
| **Model 1** | 1.21 (0.39~2.03) | 0.004 | Ref. | -20.25 (-55.38~14.89) | 0.259 | 6.04 (-23.69~35.77) | 0.690 | 34.47 (6.52~62.43) | 0.016 | 13.80 (4.89~22.70) | 0.002 |
| **Model 2** | 1.14 (0.30~1.98) | 0.008 | Ref. | -23.21 (-58.41~11.98) | 0.196 | 0.71 (-29.20~30.62) | 0.963 | 30.77 (2.35~59.19) | 0.034 | 12.51 (3.43~21.60) | 0.007 |
| **Model 3** | 1.91 (0.16~3.65) | 0.032 | Ref. | -17.40 (-52.96~18.16) | 0.338 | 9.66 (-24.16~43.48) | 0.576 | 51.99 (7.97~96.01) | 0.021 | 15.59 (1.36~29.83) | 0.032 |

Note: 95%CI, 95% Confidence interval

Model 1: Adjusted for age, sex, and race/ethnicity.

Model 2: Adjusted for the items in Model 1 + BMI, marital status, PIR, education level, alcohol consumption, and PA.

Model 3: Adjusted for the items Model 2 + diabetes, hypertension, eGFR, CVD, cancer, and the years of smoking.

**Abbreviations:** BMI, body mass index; PIR, Ratio of family income to poverty; PA, physical activity; CVD, cardiovascular disease.
